# Effects of dietary phytoestrogens on plasma testosterone and triiodothyronine (T_3_) levels in male goat kids

**DOI:** 10.1186/1751-0147-51-51

**Published:** 2009-12-10

**Authors:** David Gunnarsson, Gunnar Selstam, Yvonne Ridderstråle, Lena Holm, Elisabeth Ekstedt, Andrzej Madej

**Affiliations:** 1 Department of Molecular Biology, Umeå University, S-901 87 Umeå, Sweden; 2 Department of Anatomy, Physiology and Biochemistry, Swedish University of Agricultural Sciences, Box 7011, S-750 07 Uppsala, Sweden

## Abstract

**Background:**

Exposure to xenoestrogens in humans and animals has gained increasing attention due to the effects of these compounds on reproduction. The present study was undertaken to investigate the influence of low-dose dietary phytoestrogen exposure, i.e. a mixture of genistein, daidzein, biochanin A and formononetin, on the establishment of testosterone production during puberty in male goat kids.

**Methods:**

Goat kids at the age of 3 months received either a standard diet or a diet supplemented with phytoestrogens (3 - 4 mg/kg/day) for ~3 months. Plasma testosterone and total and free triiodothyronine (T_3_) concentrations were determined weekly. Testicular levels of testosterone and cAMP were measured at the end of the experiment. Repeated measurement analysis of variance using the MIXED procedure on the generated averages, according to the Statistical Analysis System program package (Release 6.12, 1996, SAS Institute Inc., Cary, NC, USA) was carried out.

**Results:**

No significant difference in plasma testosterone concentration between the groups was detected during the first 7 weeks. However, at the age of 5 months (i.e. October 1, week 8) phytoestrogen-treated animals showed significantly higher testosterone concentrations than control animals (37.5 nmol/l vs 19.1 nmol/l). This elevation was preceded by a rise in plasma total T_3 _that occurred on September 17 (week 6). A slightly higher concentration of free T_3 _was detected in the phytoestrogen group at the same time point, but it was not until October 8 and 15 (week 9 and 10) that a significant difference was found between the groups. At the termination of the experiment, testicular cAMP levels were significantly lower in goats fed a phytoestrogen-supplemented diet. Phytoestrogen-fed animals also had lower plasma and testicular testosterone concentrations, but these differences were not statistically significant.

**Conclusion:**

Our findings suggest that phytoestrogens can stimulate testosterone synthesis during puberty in male goats by increasing the secretion of T_3_; a hormone known to stimulate Leydig cell steroidogenesis. It is possible that feedback signalling underlies the tendency towards decreased steroid production at the end of the experiment.

## Background

Phytoestrogens are non-steroidal, diphenolic plant substances that have the capacity to bind to estrogen receptors (ERs) [1-3]. They have been suggested to protect against cancer, cardiovascular disease and osteoporosis [4]. The substances investigated in this study, i.e. genistein, daidzein, biochanin A and formononetin, belong to the isoflavone class of phytoestrogens. Isoflavones, which are found in high concentrations in soy and clover, are thoroughly studied with regard to estrogenic activity [5-8]. In rats, exposure to isoflavones during fetal/neonatal life as well as puberty may affect reproductive function. Wisniewski and colleagues found that the male offspring of rats fed a diet containing 5 mg/kg genistein, during gestation and lactation, had shortened anogenital distance (AGD), delayed pubertal onset and reduced testosterone concentrations in adulthood [9]. Consistent with this, Pan and collaborators reported that daidzein exposure during puberty impaired erectile function and lowered plasma testosterone concentrations in adulthood [10]. Additionally, genistein inhibits basal and hCG-induced Leydig cell testosterone synthesis *in vitro*, possibly by down-regulating the expression of p450scc [11,12].

A limited number of studies have been devoted to the effects of phytoestrogens on human reproductive health. These investigations indicate that adult exposure to phytoestrogens has no effects on pituitary hormone concentrations and semen parameters, but possibly lowers testosterone concentrations in the male [13-15]. In girls, phytoestrogens have been proposed to influence pubertal development. Wolff and colleagues reported a significant inverse correlation between urinary isoflavone concentrations (daidzein and genistein) and breast development in 9-year-old American girls [16].

The aim of this work was to examine the effects of low-dose dietary phytoestrogen exposure on the establishment of testosterone production during puberty in male goat kids. For this purpose, goat kids at the age of 3 months received ~3-4 mg/kg/day isoflavones (61% biochanin A, 20% formononetin, 10% genistein and 9% daidzein) for a time period of ~3 months. Phytoestrogens have been described to enhance the secretion of triiodothyronine (T_3_), which has a direct effect on Leydig cell steroidogenesis [17-19]. For this reason, we also determined plasma total and free T_3 _concentrations during the experimental period.

## Materials and methods

### Animal handling and experimental design

The experimental design and animal care were approved by the Local Animal Ethics Committee in Uppsala, Sweden. Eight male goat kids of Swedish Landrace, born in May, were used. All animals were housed in a group. Concentrate with minerals was provided twice a day at 0700 h and 1500 h; hay and water was available at all times. Four animals received two Novogen Redclover tablets/day containing 40 mg phytoestrogens (4 mg genistein, 3.5 mg daidzein, 24.5 mg biochanin A and 8 mg formononetin) (Novogen Limited Castle Hill House, UK) and four animals received two placebo tablets/day between August 19 and September 10. From September 11 until November 7 the kids were given either three Novogen Redclover tablets/day or three placebo tablets/day, to maintain the phytoestrogen concentration. Blood samples were collected once a week (except from August 28 to September 16) between August 13 and November 5 by jugular venipuncture into EDTA vacutainer tubes. From August 28 to September 16 one blood sample was collected (on September 6). On November 8, the animals were euthanized and the testes were saved for subsequent analyses of testosterone concentrations and cAMP levels.

### Determination of testosterone

Plasma and testicular testosterone concentrations were determined using the commercially available Coat-a-Count kit (Diagnostic Products Corporation, CA, USA). Prior to testosterone determination, testicular steroids were extracted by placing pieces of testes in ethanol for 14 days. Tissue was removed, ethanol evaporated to dryness with nitrogen and reconstituted in assay buffer. Serial dilutions of goat plasma with high concentrations of testosterone produced displacement curves parallel to the standard curve. The sensitivity of the testosterone assay was 0.14 nmol/l. The inter-assay coefficient of variation for quality control samples was below 10%. The corresponding intra-assay coefficient of variation was below 10% for concentrations of testosterone up to 55 nmol/l.

### Determination of total and free triiodothyronine (T_3_)

Plasma concentrations of total and free T_3 _were determined using the commercially available Coat-a-Count kit (Diagnostic Products Corporation, CA, USA). Serial dilutions of goat plasma with high concentrations of total T_3 _produced displacement curves parallel to the standard curve. The sensitivity of the total T_3 _assay was 0.14 nmol/l. The inter-assay coefficient of variation for quality control samples was 5.9%. The corresponding intra-assay coefficient of variation was below 10% for concentrations of T_3 _up to 9.22 nmol/l. The sensitivity of the free T_3 _assay was 0.3 pmol/l. The inter-assay coefficient of variation for quality control samples was 9%. The corresponding intra-assay coefficient of variation was below 10% for concentrations of T_3 _up to 72.2 pmol/l.

### cAMP analysis

Testicular cAMP concentrations were measured as described earlier [20]. In short, 150 mg of testicular tissue was homogenized in 1 ml 4 mM EDTA. The sample was then heated in boiling water for 10 min, which was followed by centrifugation at 12000 rpm for 10 min. 20 μl of the supernatant was used for determination of cAMP level. The cAMP levels were determined using a Cyclic AMP (^3^H) assay system (Amersham Pharmacia Biotech), according to the manufacturers instructions.

### Statistics

Values are presented as mean ± SEM. Repeated measurement analysis of variance using the MIXED procedure on the generated averages, according to the Statistical Analysis System program package (Release 6.12, 1996, SAS Institute Inc., Cary, NC, USA) was carried out. The statistical model included dose (two groups), time (twelve weeks), interaction between dose and time, and the random effect of goats within a group.

## Results

Semen evaluation and sexual behaviour indicated that all male goats had reached sexual maturity on October 25. All data from one goat kid were removed from the evaluation due to a bilateral sperm granuloma, which was found at autopsy.

### Plasma testosterone concentrations

**Figure 1:**
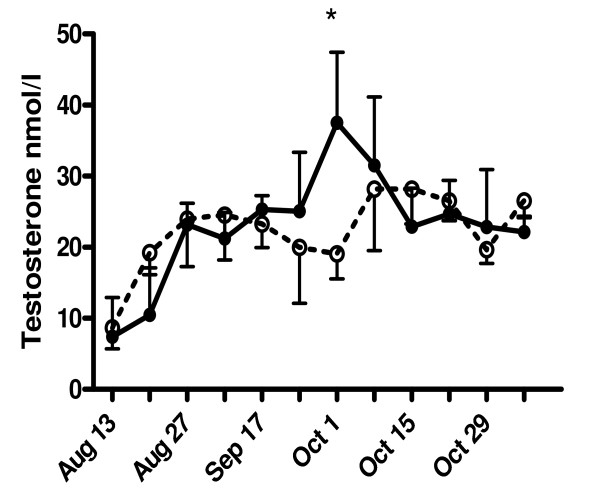
**Effects of phytoestrogens on the plasma testosterone concentrations in male goat kids**. Goat kids at the age of 3 months received either a standard diet (controls) or a diet supplemented with phytoestrogens (3 - 4 mg/kg/day) for a period of ~3 months (August 19 to November 7). At week 8 of the experiment (i.e. October 1), phytoestrogen-exposed animals (closed circles, solid line) had significantly (* P < 0.05) higher testosterone concentrations than controls (open circles, dashed line).

As shown in Fig. 1, the only time point at which a significant difference between controls and phytoestrogen-treated animals could be detected was at week 8 (October 1) of the experiment (19.1 ± 5.2 nmol/l vs 37.5 ± 6.0 nmol/l). At the end of experiment, the mean testosterone levels were slightly lower (no significant difference) in phytoestrogen-treated goats.

### Plasma free and total triiodothyronine (T_3_) concentrations

**Figure 2:**
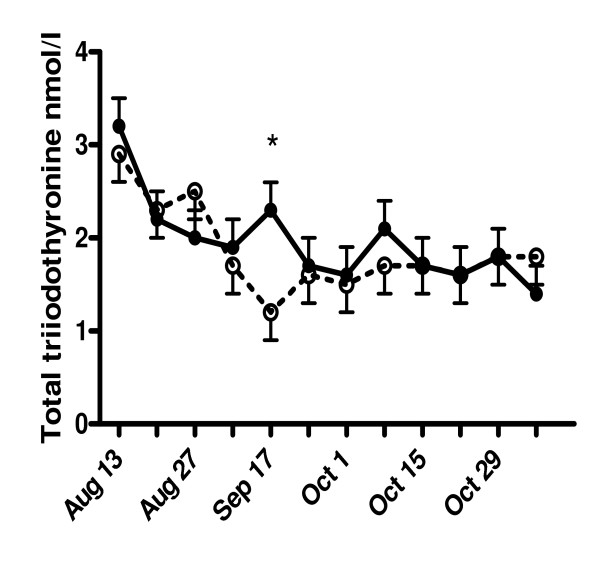
**Effects of phytoestrogens on plasma total triiodothyronine (T_3_) concentrations in male goat kids**. Goat kids at the age of 3 months received either a standard diet (controls) or a diet supplemented with phytoestrogens (3 - 4 mg/kg/day) for a period of ~3 months (August 19 to November 7). At week 6 of the experiment (i.e. September 17), phytoestrogen-exposed animals (closed circles, solid line) had significantly (* P < 0.05) higher total T_3 _concentrations than controls (open circles, dashed line).

**Figure 3:**
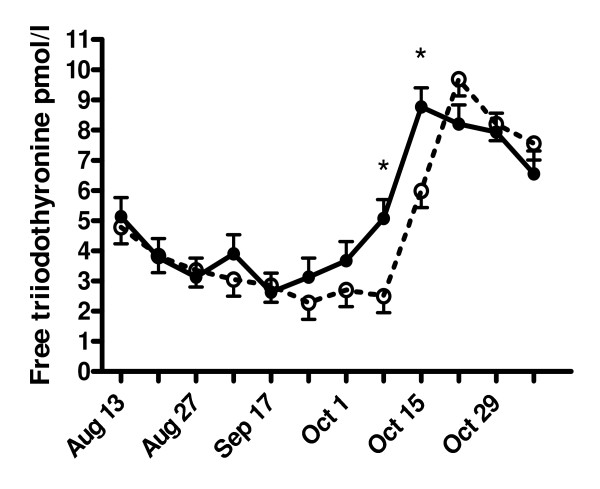
**Effects of phytoestrogens on the plasma free triiodothyronine (T_3_) concentrations in male goat kids**. Goat kids at the age of 3 months received either a standard diet (controls) or a diet supplemented with phytoestrogens (3 - 4 mg/kg/day) for a period of ~3 months (August 19 to November 7). At week 9 and 10 of the experiment (i.e. October 8 and 15), phytoestrogen-exposed animals (closed circles, solid line) had significantly (* P < 0.05) higher free T_3 _concentrations than controls (open circles, dashed line).

The total T_3 _levels gradually decreased from around 3 nmol/l in the beginning of the experiment to around 1.8 nmol/l three weeks later in both groups (Fig. 2). At week 6 of the experiment (i.e. September 17) total T_3 _levels were significantly higher in the phytoestrogen group than in the control group (2.3 ± 0.3 vs. 1.2 ± 0.2 nmol/l). The free T_3 _levels at weeks 9 and 10 (i.e. October 8 and 15) were significantly higher in treated animals than in controls (5.1 ± 0.6 vs. 2.5 ± 0.6 pmol/l and 8.8 ± 0.6 vs. 6.0 ± 0.6 pmol/l, respectively) (Fig. 3).

### Testicular testosterone concentrations

**Figure 4:**
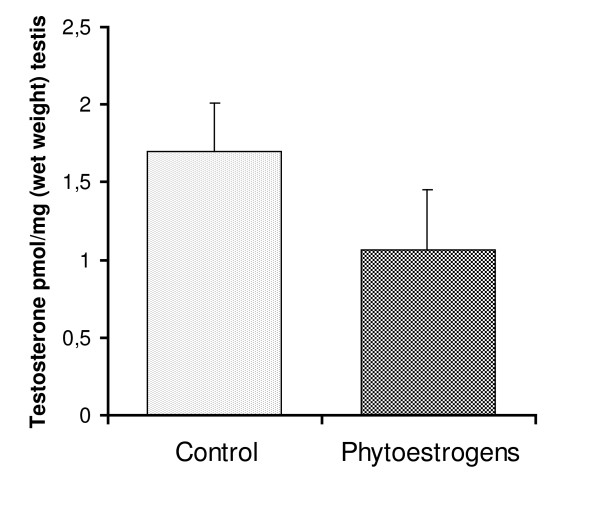
**Testicular testosterone concentrations in goat kids at the end of the experiment**. Goat kids at the age of 3 months received either a standard diet (controls) or a diet supplemented with phytoestrogens (3 - 4 mg/kg/day) for a period of ~3 months (August 19 to November 7). Data are expressed as mean ± S.E.M.

Testicular testosterone concentrations were measured at the end of the experiment. No significant difference was found between phytoestrogen-fed animals and controls (Fig. 4).

### Testicular cAMP concentrations

**Figure 5:**
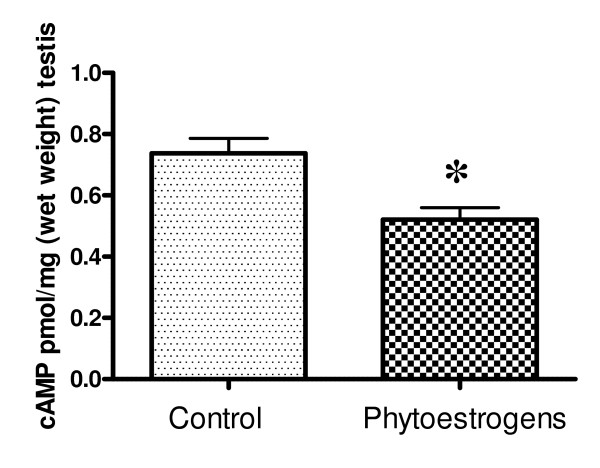
**Testicular cAMP concentrations in goat kids at the end of the experiment**. Goat kids at the age of 3 months received either a standard diet (controls) or a diet supplemented with phytoestrogens (3 - 4 mg/kg/day) for a period of ~3 months (August 19 to November 7). Data are expressed as mean ± S.E.M. * P < 0.05.

Testicular cAMP concentrations were determined at the end of the experiment. As shown in Fig. 5, the concentration of cAMP was significantly lower (~25%) in phytoestrogen-treated animals than in controls.

## Discussion

This study shows that phytoestrogens can stimulate testosterone synthesis during puberty in male goats. The rise in testosterone concentration was preceded by an elevation of plasma total T_3_, suggesting that phytoestrogens exerts their stimulatory effect on steroidogenesis by increasing the secretion of T_3_, a hormone known to increase Leydig cell testosterone synthesis.

Most previous studies have reported suppressive effects of phytoestrogens on testosterone production. Perinatal as well as pubertal exposure to isoflavones has been found to decrease plasma testosterone levels [9,10]. It is possible that the discrepancy between our study and previous ones is due to the different doses used. Almstrup and colleagues demonstrated that most phytoestrogens are aromatase inhibitors at low concentrations but estrogenic at higher concentrations [21]. They found that biochanin A and formononetin were aromatase inhibitors at concentrations < 1 μM, with biochanin A being the more potent of the two. Hence, it is plausible that the low dose treatment used in our study causes an inhibition of aromatase activity and a subsequent elevation of testosterone.

The fact that the tablets used in the present experiment contained approximately 60% biochanin A supports this hypothesis.

However, since plasma concentrations of total and free T_3 _were significantly higher in phytoestrogen-fed animals than controls, it seems likely that the major mechanism underlying the increased testosterone production was a stimulation of T_3 _secretion. It is now generally accepted that T_3 _has an important role in the regulation of Leydig cell differentiation and function [22].

Hypothyroidism is associated with reduced testosterone synthesis and *in vitro *studies have revealed that T_3 _has a direct stimulatory effect on Leydig cell steroidogenesis in several species, including mouse, rat and goat [18,23-25]. Interestingly, Maran and co-workers described a stimulation of testosterone production in Leydig cells isolated from pubertal rats [18]. The exact mechanism whereby T_3 _induces steroidogenesis remains to be established, but an important role of StAR has been demonstrated [25]. The hypothesis of T_3 _being involved in phytoestrogen-induced increment of testosterone is strengthened by previous reports from our laboratory and others showing an increased T_3 _secretion after phytoestrogen exposure in animals as well as humans [17,19].

However, other studies have concluded that isoflavones do not affect thyroid hormones in humans. Dietary exposure to 1-3 mg/kg/day isoflavones has been found not to alter plasma concentrations of thyroid stimulating hormone (TSH), thyroxine (T_4_) or T_3 _in adult men as well as postmenopausal women [26-28]. However, direct comparisons are difficult since the studies mentioned above used soy protein isolates, which contain a mixture of isoflavones with different properties. Genistein is typically the main isoflavone found in soy protein isolates, whereas the present study and others showing increased T_3 _secretion have used formulas containing predominantly other isoflavones, i.e. biochanin A and daidzein [17,19].

It is known that isoflavones have different properties, with regard to ER binding and aromatase inhibition. For example, genistein has a significantly higher affinity for ERs than biochanin A [29,30]. Biochanin A, on the other hand, is an efficient aromatase inhibitor, whereas genistein does not inhibit this enzyme [21,31]. Hence, it is not surprising that exposure to different mixtures of isoflavones induce very different effects.

In addition, the outcome of phytoestrogen treatment is dependent on the timing of exposure. Levy and collaborators found that *in utero *exposure to genistein delayed pubertal onset in female rats, whereas Kouki and colleagues reported the opposite effect after lactational exposure [32,33]. It is possible that the increment in T_3 _and testosterone found in the present study is restricted to the pubertal period. Indeed, there were no significant differences in these parameters between phytoestrogen-fed animals and controls when they reached sexual maturity. Altered androgen synthesis may influence pubertal development and increased testosterone concentrations have been associated with advanced pubertal onset after exposure to other endocrine disruptors [34].

At the end of the experiment we observed a tendency towards reduced steroid synthesis in phytoestrogen-fed goats. Although neither testicular nor plasma testosterone concentrations differed significantly between the groups, mean values were lower in the phytoestrogen group. In addition, phytoestrogen-treated animals had significantly lower testicular cAMP concentrations; an observation previously associated with decreased steroidogenesis [20]. However, since Leydig cells are not the only testicular cell type dependent on cAMP signalling this result should be interpreted with some caution. It is possible that negative feedback signalling underlies the (suspected) inhibition of testosterone synthesis.

The discrepancy between our findings after exposure to isoflavones present in clover and experiments using soy protein isolates indicates that traditional soy based diets are safe (in this respect), whereas red clover extracts may influence thyroid hormone release and steroid synthesis at low doses. The dose used in the present study (3-4 mg/kg/day) is only 3-4 fold higher than the one in red clover extracts consumed by menopausal women. The intake in clover-grazing animals may well exceed 3-4 mg/kg/day. For this reason, future studies should address the influence of low-dose exposure to red clover isoflavones on the endocrine system in women as well as grazing animals. In addition, future experiments should be designed to further characterize the mechanisms of action of each isoflavone compound.

## Conclusions

This study demonstrates that low-dose dietary phytoestrogen exposure may stimulate testosterone synthesis and T_3 _secretion in pubertal male goats.

## Competing interests

The authors declare that they have no competing interests.

## Authors' contributions

Sampling was carried out at the Swedish University of Agricultural Sciences (EE, YR, LH). Experimental analyses and writing of the manuscript were done jointly by the Swedish University of Agricultural Sciences (AM, EE, LH) and Umeå University (DG, GS). All authors read and approved the final manuscript.
